# Fabrication and Characterization of Flexible pH Sensors Based on Pulsed Laser-Ablated Graphene/MoS_2_ Interdigitated Electrodes

**DOI:** 10.3390/nano15141115

**Published:** 2025-07-18

**Authors:** Zhaochi Chen, Chengche Liu, Minh-Quang Tran

**Affiliations:** 1 Graduate Institute of Biomedical Optomechatronics, Taipei Medical University, Taipei 230, Taiwan; 2 Department of Physiology and Biophysics, Graduate Institute of Physiology, National Defense Medical Center, Taipei 114, Taiwan; chencheliu2002@gmail.com; 3 Department of Mechanical Engineering, TUETECH University, Thai Nguyen 250000, Vietnam; tranminhquang_ck@tuetech.edu.vn

**Keywords:** pulsed laser ablation, graphene/MoS_2_ composites thin film, IDEs, pH sensor

## Abstract

Point-of-care (POC) diagnostic technologies have become essential for the real-time monitoring and management of chronic wounds, where maintaining a moist environment and controlling pH levels are critical for effective healing. In this study, a flexible pH sensor based on a graphene/molybdenum disulfide (graphene/MoS_2_) composite interdigitated electrode (IDE) structure was fabricated using pulsed laser ablation. The pH sensor, with an active area of 30 mm × 30 mm, exhibited good adhesion to the polyethylene terephthalate (PET) substrate and maintained structural integrity under repeated bending cycles. Precise ablation was achieved under optimized conditions of 4.35 J/cm^2^ laser fluence, a repetition rate of 300 kHz, and a scanning speed of 500 mm/s, enabling the formation of defect-free IDE arrays without substrate damage. The influence of laser processing parameters on the surface morphology, electrical conductivity, and wettability of the composite thin films was systematically characterized. The fabricated pH sensor exhibited high sensitivity (~4.7% change in current per pH unit) across the pH 2–10 range, rapid response within ~5.2 s, and excellent mechanical stability under 100 bending cycles with negligible performance degradation. Moreover, the sensor retained > 95% of its stable sensitivity after 7 days of ambient storage. Furthermore, the pH response behavior was evaluated for electrode structures with different pitches, demonstrating that structural design parameters critically impact sensing performance. These results offer valuable insights into the scalable fabrication of flexible, wearable pH sensors, with promising applications in wound monitoring and personalized healthcare systems.

## 1. Introduction

Chronic wounds, such as diabetic ulcers and pressure sores, pose significant challenges to healthcare systems worldwide due to their prolonged healing times and susceptibility to infections [1,2]. Effective management of these wounds necessitates real-time monitoring of key biomarkers, notably pH levels, which can provide insights into the wound healing process and early signs of infection [3,4]. Traditional wound assessment methods are often subjective and lack the capability for continuous monitoring, underscoring the need for advanced diagnostic tools [5]. The World Union of Wound Healing Societies has emphasized the importance of integrating such diagnostic tools into clinical and home-care settings to enhance patient outcomes [6]. In particular, point-of-care (POC) pH sensors offer a promising solution by enabling immediate, on-site analysis, thereby facilitating timely interventions and personalized treatment strategies [7,8].

Despite the potential benefits, existing pH sensors face several limitations that hinder their widespread adoption in wound care applications. Conventional glass electrode pH sensors are rigid, fragile, and unsuitable for conformal contact with the irregular surfaces of wounds [9,10]. Moreover, they often require frequent calibration and are not designed for continuous monitoring. Recent advancements have explored flexible and wearable pH sensors; however, issues such as limited sensitivity, poor durability under mechanical stress, and complex fabrication processes persist [11,12,13]. Additionally, ensuring biological wettability and improving operational stability remain critical challenges. Addressing these issues is essential for developing reliable, user-friendly pH sensors suitable for real-time wound monitoring in diverse settings [14,15].

Graphene has emerged as one of the most promising materials for electrochemical sensors owing to its extraordinary electrical conductivity, large specific surface area, chemical stability, and mechanical flexibility [16,17,18,19]. Recent studies have demonstrated that graphene-based electrodes exhibit enhanced charge transfer kinetics, low signal-to-noise and excellent adaptability to flexible substrates, enabling their application in various biosensing platforms [20,21]. The integration of flexible materials enables the pH sensor to conform seamlessly to the dynamic and irregular topographies of wound sites, allowing continuous, non-invasive monitoring even during patient movement. Such flexibility minimizes signal artifacts induced by mechanical deformation and is therefore critical for applications requiring direct skin or wound contact [22,23]. Notably, the intrinsic functionalization capabilities of composite graphene structures enable their direct application in biochemical sensing without the necessity for external modification with enzymes, nucleic acids, or antibodies, thereby significantly enhancing their versatility across diverse biosensing platforms [24]. Building on these advantages, composite materials combining graphene with molybdenum disulfide (MoS_2_) have been increasingly explored to address the limitations of pristine graphene and to further enhance sensing performance [25]. MoS_2_-based composites have been widely adopted for detecting various analytes due to their layered structure and tunable electronic properties. Recent works reported MoS_2_ nanosheets-decorated SnO_2_ composites achieving high sensitivity for CO, NH_3_, and H_2_ gases sensing [26], and 2D MoS_2_ nanoparticles anchored strategy in chemiresistive gas sensors under room temperature [27]. These studies reinforce MoS_2_’s potential as a key component in electrochemical sensors.

The graphene/MoS_2_ heterostructures exhibit improved sensing responsiveness, higher electron mobility, and greater mechanical robustness under bending stress, making them highly suitable for flexible, wearable pH sensors [28]. Moreover, the integration of MoS_2_ not only boosts the sensitivity and selectivity toward pH changes but also imparts better stability under electrochemical conditions [29,30]. Sakthivel et al. fabricated large-area graphene/MoS_2_ chemiresistive sensors via spray pyrolysis, achieving a hierarchical thin-film structure with enhanced NO_2_ sensitivity (81% at 1000 ppm), rapid response (12 s), and improved stability compared to individual MoS_2_ with graphene films, while also investigating the effects of humidity and sensing mechanisms [31]. Guo et al. developed a high-sensitivity humidity sensor based on MoS_2_/graphene oxide quantum dot (GOQD) composite films deposited on Au interdigitated electrodes (IDEs) via a drop coating method, achieving a sensitivity of 369 pF/%RH approximately twenty times higher than that of pure MoS_2_ sensors along with rapid response, low hysteresis, and stable long-term performance [32].

Emerging fabrication techniques, particularly pulsed laser ablation, offer promising avenues for developing advanced pH sensors. Pulsed laser ablation enables precise, maskless patterning of conductive materials on flexible substrates without the need for chemical reagents, thereby simplifying the manufacturing process [33,34]. The technique minimizes thermal damage to the substrate, allowing the integration of sensitive materials like graphene and its composite structures [35]. The synergy between pulsed laser fabrication and graphene-based composites holds significant potential for creating flexible, high-performance pH sensors tailored for wound monitoring [36,37]. Chhetry et al. demonstrated the effectiveness of laser-induced fabrication for ablating MoS_2_-decorated graphene composite electrodes by one-step carbonization of MoS_2_-coated polyimide films. The laser ablation process enabled the formation of three-dimensional porous graphene nanoflakes uniformly decorated with MoS_2_, yielding stable electrical properties and robust mechanical integrity. The resulting sensor exhibited outstanding piezoresistive performance, including a high gauge factor (GF ≈ 1242), minimal hysteresis (~2.75%), an ultralow detection limit (0.025%), and rapid relaxation (~0.17 s), with durable functionality over 12,000 strain–release cycles. Furthermore, the composite electrodes facilitated the precise detection of subtle physiological signals, highlighting the potential of laser ablation graphene/MoS_2_ structures for high-performance wearable sensing applications [38]. Jeon et al. demonstrated the effective use of laser ablation to fabricate vertically stacked graphene/MoS_2_ heterostructures at room temperature, overcoming the thermal damage issues inherent to conventional chemical vapor deposition processes. By utilizing the distinct photothermal absorption characteristics of graphene and MoS_2_, the laser ablation technique enabled precise, localized synthesis of the heterojunction without compromising the functionality of adjacent materials. The resulting composite electrodes exhibited stable electrical performance and reliable photoresponse behavior, validating the potential of laser ablation as a scalable and material-preserving method for two-dimensional semiconductor device fabrication. This approach opens new avenues for the development of sensors using laser-processed graphene/MoS_2_ composites [39].

In this study, we demonstrated a flexible and wearable pH sensor leveraging graphene/MoS_2_ composite IDE fabricated via pulsed laser ablation. Particular emphasis was placed on optimizing the laser processing parameters, including fluence, repetition rate, and scanning speed, enabling precise, defect-free patterning of IDE structures without compromising the integrity of the polyethylene terephthalate (PET) substrate. We characterized the electrochemical performance, mechanical durability under repeated bending cycles, and pH sensitivity across varying IDE pairs which were evaluated. This study was carried out to validate the feasibility of the sensor for instant, tunable, and non-invasive pH monitoring, addressing the need for real-time biomarker-free tracking in dynamic wound environments. By bridging advanced material fabrication methodologies with clinically relevant sensing capabilities, this study seeks to contribute toward the realization of next-generation smart wound care systems, facilitating improved patient outcomes and personalized healthcare interventions.

## 2. Materials and Methods

### 2.1. Preparation of Graphene/MoS_2_ Composite Thin Films on PET Substrates

**Figure 1:**
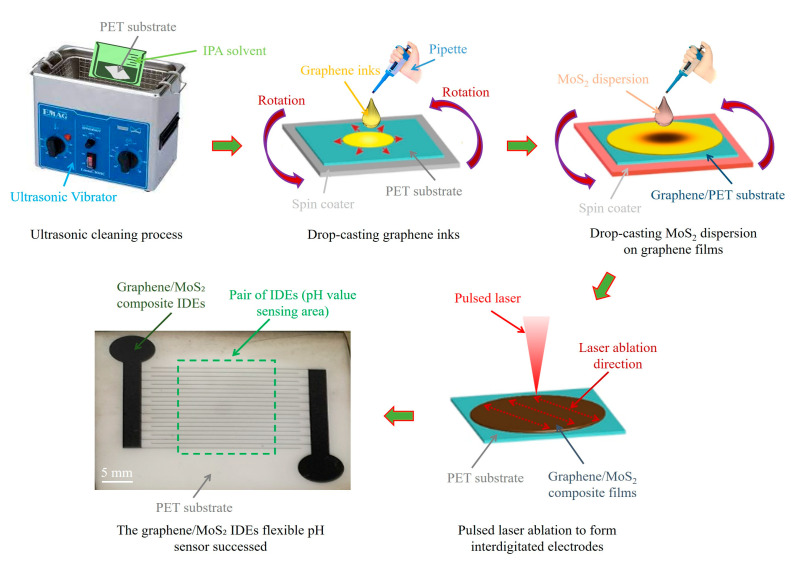
Schematic illustration of the fabrication process for the flexible graphene/MoS_2_ IDEs pH sensor.

The fabrication of graphene/MoS_2_ composite thin films was performed using a two-step drop-casting process. Initially, flexible polyethylene terephthalate (PET) substrates (thickness: 175 μm) were cut into 30 mm × 30 mm squares and ultrasonically cleaned in isopropyl alcohol (IPA) and deionized (DI) water for 10 min each to eliminate surface contaminants. Graphene ink (Model: 808261, sheet resistance: 10 Ω/sq at 25 μm thickness, particle size ~1 μm, Sigma-Aldrich, St. Louis, MO, USA) was drop-cast onto the cleaned PET substrate at 1000 rpm for 20 s. The coated graphene layer was thermally cured at 80 °C for 1.5 h, forming a uniform film with a thickness of 30 ± 3 μm. For the MoS_2_ layer, 150 mg of MoS_2_ powder (Model: 69860, particle size ~6 μm, Sigma-Aldrich, St. Louis, MO, USA) was dispersed in 20 mL of a 45% ethanol–water mixture (ethanol/water = 45:55, *w*/*w*). This solvent system was selected due to its moderate polarity and pH (~6.3), which enhances the dispersion stability of MoS_2_ nanosheets by reducing particle aggregation and promoting better wetting on the graphene surface [40]. Ultrasonication for 90 min ensured uniform dispersion of MoS_2_ particles. The resulting suspension was centrifuged at 1000 rpm for 15 min, and the upper one-third of the supernatant was drop-cast onto the preformed graphene/PET films. A subsequent thermal curing at 120 °C for 2 h facilitated solvent removal and interfacial adhesion, resulting in graphene/MoS_2_ composite films with an average total thickness of 40 ± 4 μm, as shown in Figure 1. The final thickness (~40 ± 4 μm) of the graphene/MoS_2_ composite film was controlled by modulating the drop-casting volume (20 µL per deposition), spin rate (1000 rpm for 20 s), and subsequent curing at 120 °C for 2 h. Previous studies demonstrated that such multi-step drop-casting combined with thermal curing enables uniform thin films for flexible electronics [41]. The ethanol–water mixture’s near-neutral pH not only prevents potential surface oxidation of MoS_2_ during deposition but also supports the functional integrity of the composite film for reliable physiological pH sensing across a wide range (pH 2–10) [42].

### 2.2. Design of IDEs Electrode Geometry

The geometric configuration of IDEs has a profound impact on the electrochemical performance of sensors, particularly regarding detection sensitivity and signal fidelity. Previous studies have emphasized that IDE parameters such as electrode pair numbers, spacing, and width critically influence electric field distribution and analyte interaction. For instance, Mathur et al. demonstrated that variations in the gap of Au-coated IDEs directly modulate electrochemical sensitivity, underscoring the necessity of precise geometric optimization in IDE design [43]. Similarly, these studies reported that while increasing IDE density enhances surface area and field strength, excessive density may introduce fabrication challenges and undesirable parasitic effects, such as signal saturation and electrical cross-talk [44,45]. In this study, three IDE configurations were fabricated to examine the relationship between electrode geometry and pH sensing performance. Each configuration consists of alternating graphene/MoS_2_ composite conductive films with specified width and IDE spacing, including the number of electrode pairs and the corresponding pitch, which are summarized in Table 1. These patterned electrode arrays were subsequently employed as active sensing interfaces for pH measurements and electrical characterization.

### 2.3. Laser Ablation Process Optimization for Patterning Graphene/MoS_2_ Composite IDEs

**Figure 2:**
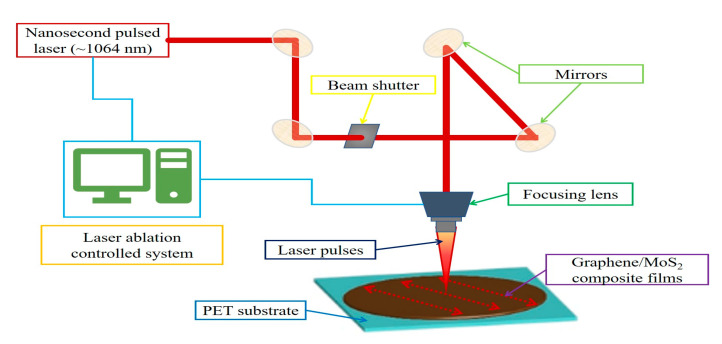
Schematic diagram of the nanosecond laser ablation system used for patterning graphene/MoS_2_ composite thin films.

To achieve precise patterning of IDEs on graphene/MoS_2_ composite films, nanosecond pulsed laser ablation was employed. The laser system utilized was a fiber laser (IPG Photonics Co., Oxford, MA, USA) operating at a wavelength of 1064 nm with a pulse duration of 200 ns and a maximum average power of 22 W. Throughout the ablation experiments, the scanning speed was maintained at 500 mm/s, the pulse repetition rate at 300 kHz, and each area underwent three passes to ensure consistent films removal. Prior to ablation, a power meter was used to calibrate the laser output energy at various attenuation settings. Here, the ablation fluence (*F*, J/cm^2^) and single-pulse energy (*E*, J) were estimated using the following Equations (1) and (2) [46]:

(1) $$F=\frac{4E}{\begin{array}{c} {\pi d}^{2} \end{array}}$$

(2) $$D^{2}=2w_{0}^{2}ln(\frac{F}{\begin{array}{c} \begin{array}{c} F_{th}(N) \end{array} \end{array}})$$

where *d* is the laser beam diameter (μm), *D* is the laser ablation width (μm), *w*_0_ is the laser beam waist (μm), *F_th_* is the laser ablation threshold (J/cm^2^). These calculations are critical for determining the appropriate laser parameters to achieve efficient ablation without damaging the underlying substrate. The incubation effect under pulsed laser irradiation leads to a pronounced reduction in the ablation threshold as the cumulative pulse energy increases. Beyond a critical number of pulses, the threshold energy density reaches a saturation regime. The resulting ablation width and structural quality exhibit a strong dependence on the applied fluence, highlighting the critical need to optimize energy density parameters to achieve a balance between material removal efficiency and morphological precision [47]. The experimental setup, including the self-assembly laser system configuration and ablation process flow, is shown in Figure 2.

### 2.4. Electrochemical Configuration for pH Measurement

**Figure 3:**
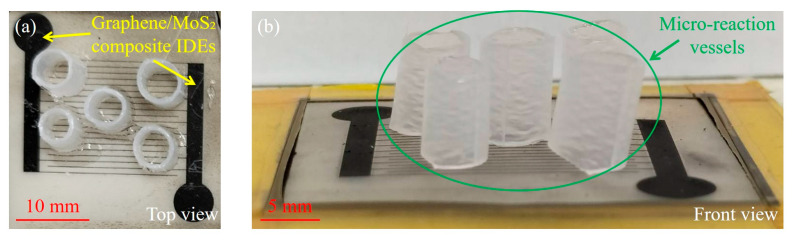
Photographic images of the pH sensing platform integrated with graphene/MoS_2_ composite IDEs: (**a**) top view; (**b**) side view.

To evaluate the pH-responsive behavior of the graphene/MoS_2_ composite IDEs, a constant bias voltage of 1 V was applied across the sensing interface, and the resulting current was recorded to assess the corresponding electrical response. The measurement setup comprised parallel IDEs interfaced with multiple micro-reaction vessels arranged on a spatially staggered platform, as shown in Figure 3. Each vessel was individually filled with pH buffer solutions ranging from pH 2 to pH 10 using a calibrated micropipette. The real-time current changes were continuously monitored during solution contact with the IDEs surface, enabling simultaneous multi-site pH assessment and calibration of the sensing IDEs.

### 2.5. Characterization of Graphene/MoS_2_ Composite Film Properties

The structural, electrical, and interfacial properties of the laser-ablated graphene/MoS_2_ IDEs were systematically characterized. Surface morphology was investigated using laser scanning confocal microscopy (LSCM, LEXT OLS5100, Olympus, Shinjuku, Tokyo, Japan) and scanning electron microscopy (SEM, JEOL JSM-7610F, Akishima, Tokyo, Japan) to assess topographical features and ablation fidelity. Electrical performance was evaluated via current–voltage (*I–V*) measurements to determine composite films resistance. The sensing capability was further examined through pH-dependent response analysis, while the wettability of the film was assessed by static contact angle (CA) measurements. Additionally, Raman spectroscopy (Nanofinder FLEX, Tokyo Instruments, Inc., Suginami, Tokyo, Japan) was employed to analyze the chemical composition and characteristics of the composite films, allowing the identification of phase alterations induced by the laser ablation. Collectively, these characterization results provide critical insights into the influence of varying laser fluence on film ablation efficiency, electrode integrity, and overall sensor performance.

## 3. Results and Discussion

### 3.1. Laser Ablation Characteristics and Surface Morphology

**Figure 4:**
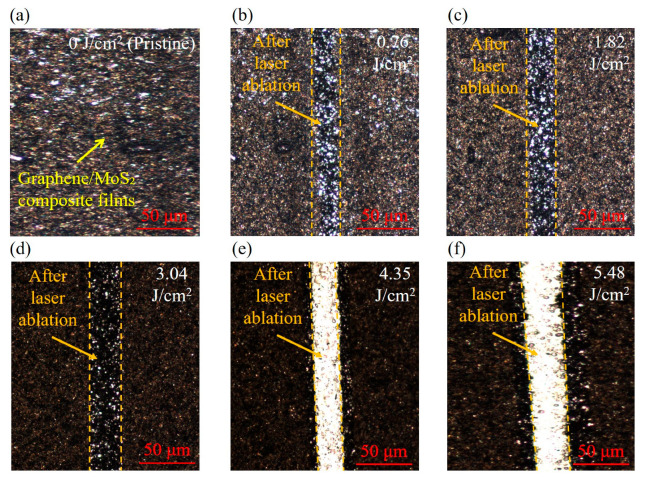
LSCM images of graphene/MoS_2_ composite films morphology subjected to pulsed laser ablation under varying fluences: (**a**) the pristine surface; (**b**) 0.76 J/cm^2^; (**c**) 1.82 J/cm^2^; (**d**) 3.04 J/cm^2^; (**e**) 4.35 J/cm^2^; (**f**) 5.48 J/cm^2^.

To ensure the effect of laser fluence on the ablation behavior of graphene/MoS_2_ composite films, a series of confocal micrographs were acquired under controlled laser ablation fluence ranging from 0 to 5.48 J/cm^2^, as shown in Figure 4. The pristine sample exhibits a uniform, densely packed film morphology without evidence of structural damage, confirming the integrity of the graphene/MoS_2_ composites prior to irradiation (see Figure 4a). Upon application of a low fluence of 0.76 J/cm^2^, partial ablation is observed along the laser trace, characterized by localized bright scattering indicative of surface decomposition or thinning (see Figure 4b). As the fluence increases to 1.82 J/cm^2^ and 3.04 J/cm^2^, more extensive disruption appears along the central ablated line, suggesting progressive decomposition and residual layered materials (see Figure 4c,d). At a fluence of 4.35 J/cm^2^, the ablation groove becomes more defined and continuous, with the contrast between ablated and non-ablated regions becoming increasingly distinct (see Figure 4e). This transition suggests the fluence surpasses the ablation threshold of the composite films, enabling cleaner removal. Further increasing the fluence to 5.48 J/cm^2^ results in highly efficient material clearance, with the ablation track appearing significantly wider and more reflective, likely due to underlying substrate exposure and thermal expansion effects. The pronounced brightness and scattering observed in these regions are consistent with complete detachment of the composite layer and potential local reflow or melting (see Figure 4f). These findings align with prior observations reported by Zhu et al., where laser-induced ablation of MoS_2_ composite films was shown to transition from surface modification to full removal with increasing laser ablation fluence, accompanied by a sharp change in optical contrast and edge definition [48]. Such energy-dependent behavior is critical for tailoring laser parameters in microelectrode patterning and flexible sensor fabrication.

**Figure 5:**
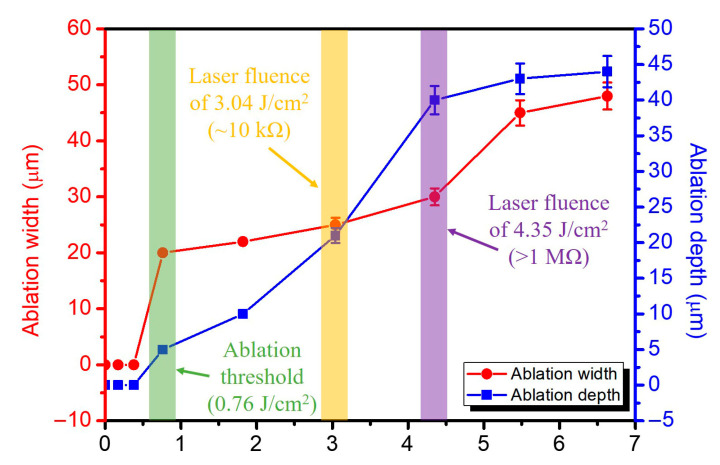
Correlation between laser fluence and both ablation width (red curve, left axis) and ablation depth (blue curve, right axis).

The relationship between the ablation width and depth at different laser fluences is shown in Figure 5. The fluence of 0.76 J/cm^2^ (green arrow) represents the minimum energy density required to initiate observable material removal on the graphene/MoS_2_ surface, corresponding to the ablation threshold. At this point, the ablation width and depth were minimal at ~20 μm and ~5 μm, respectively, and the electrical continuity remained mostly intact. This regime is governed by the incubation-assisted soft threshold mechanism, where cumulative pulse energy enables initial carbon matrix disruption without significant substrate exposure. The electrical resistance in this region remains relatively low, indicating partial retention of conductive pathways. As the laser fluence increased beyond 3.04 J/cm^2^ (yellow arrow), both the ablation width and depth exhibited a rapid rise, correlating with a transition in electrical resistance to ~10 kΩ, indicating partial electrical disruption. At a higher fluence of 4.35 J/cm^2^ (purple arrow), the electrical resistance exceeded 1 MΩ, confirming effective electrical insulation due to complete material removal and substrate exposure. This transition point marks the functional ablation regime suitable for electrode isolation in device fabrication. Ablation depth reached a saturation value of ~45 μm beyond 5.0 J/cm^2^, suggesting a plateau effect due to thermal diffusion limits and material boundary confinement. Meanwhile, the ablation width continued to increase, albeit at a reduced rate, potentially leading to edge thermal damage or surface roughening. Error bars represent the standard deviation from five repeated measurements. These findings underscore the importance of fluence optimization to ensure clean, reproducible microstructuring while maintaining device integrity.

**Figure 6:**
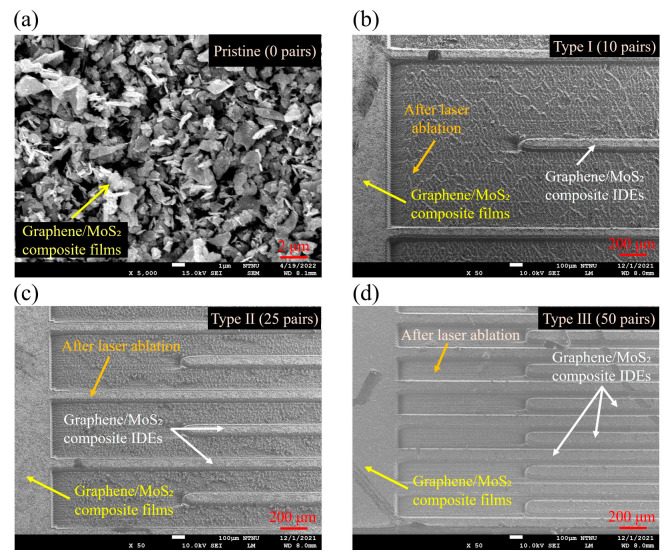
SEM images of graphene/MoS_2_ composite IDEs fabricated under various pairs at the optimized laser fluence of 4.35 J/cm^2^: (**a**) pristine (0 pairs); (**b**) 10 pairs; (**c**) 25 pairs; (**d**) 50 pairs.

The selected fluence of 4.35 J/cm^2^ was determined to be optimal based on prior analyses of ablation width, depth, and electrical isolation (see Figure 5), producing complete film removal within the irradiated regions without damaging the surrounding composite or PET substrate. Figure 6 presents representative SEM images of graphene/MoS_2_ composite IDEs fabricated at this optimized fluence. The pristine film (0 pairs) displays a dense layered structure of MoS_2_ embedded in the graphene matrix, while laser-processed Type I, II, and III IDEs were 10, 25, and 50 pairs, respectively, that show uniform, well-defined electrode geometries without visible thermal degradation. At this energy density, the ablated grooves exhibit clean boundaries and high contrast relative to unablated regions, indicating efficient photothermal material removal and strong spatial confinement of the beam. Furthermore, as the IDE pairs increased, the fidelity of the patterned electrodes remained consistent, with uniform interspacing and minimal thermal distortion across all three configurations. This confirms that laser ablation behavior enables reproducible and scalable fabrication of high-density microelectrode arrays, suitable for subsequent electrochemical sensing applications.

### 3.2. Materials Analysis and Surface Characterization

**Figure 7:**
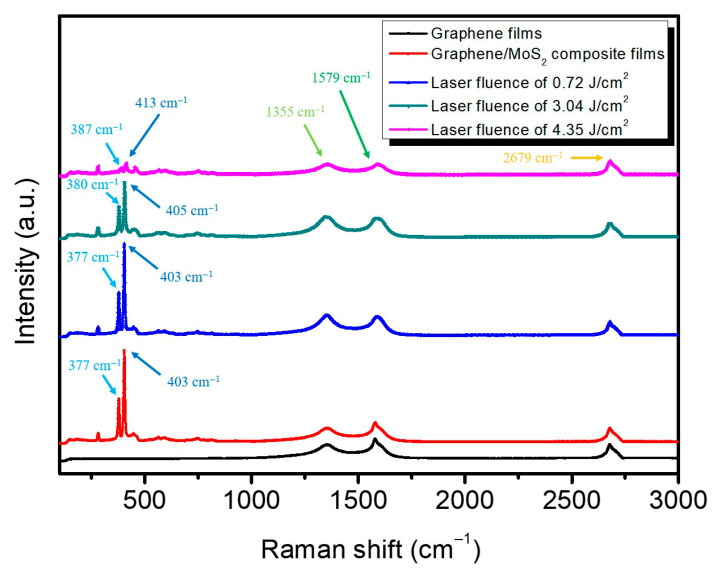
Raman spectra of graphene/MoS_2_ composite films before and after laser ablation at varying fluences.

To investigate the correlation between laser fluence and the wettability of graphene/MoS_2_ composite films, Figure 7 presents the Raman spectra of graphene/MoS_2_ composite films before and after laser ablation at varying fluences, which are 0.72 J/cm^2^, 3.04 J/cm^2^, and 4.35 J/cm^2^. The pristine graphene (graphene films) and graphene/MoS_2_ spectra (black and red curves, respectively) serve as references for post-ablation comparisons. Two prominent MoS_2_ characteristic peaks, corresponding to the *E^1^_2g_* (in-plane vibration) and *A_1g_* (out-of-plane vibration) modes are observed at ~377 cm^−1^ and ~403 cm^−1^ in the unablated graphene/MoS_2_ composite film. With increasing laser fluence, the *E^1^_2g_* peak progressively shifts from 377 cm^−1^ to 387 cm^−1^, and the *A_1g_* peak shifts from 403 cm^−1^ to 413 cm^−1^. This pronounced blue shift suggests that higher energy densities induce stronger local lattice compression and surface strain, likely due to pulsed laser-dependent photothermal dehydration and densification of the MoS_2_ layers. Such shifts are also indicative of enhanced wettability or hydration-induced strain–relaxation phenomena, consistent with earlier studies where water molecule adsorption or polar solvent interaction compresses the MoS_2_ interlayers [49]. Additionally, the graphene-related *D*, *G*, and *2D* bands are evident near 1355 cm^−1^, 1579 cm^−1^, and 2679 cm^−1^, respectively. The increased *D* band intensity and slight upshift of the *G* band at higher fluences suggest elevated defect generation and possible functional group formation, both of which improve hydrophilicity and facilitate proton conduction for electrochemical sensing. Collectively, these spectroscopic changes confirm that laser ablation modulates the structural and interfacial properties of graphene/MoS_2_ composite films, enhancing their surface polarity and wettability which are key attributes for sensitive pH detection applications.

**Figure 8:**
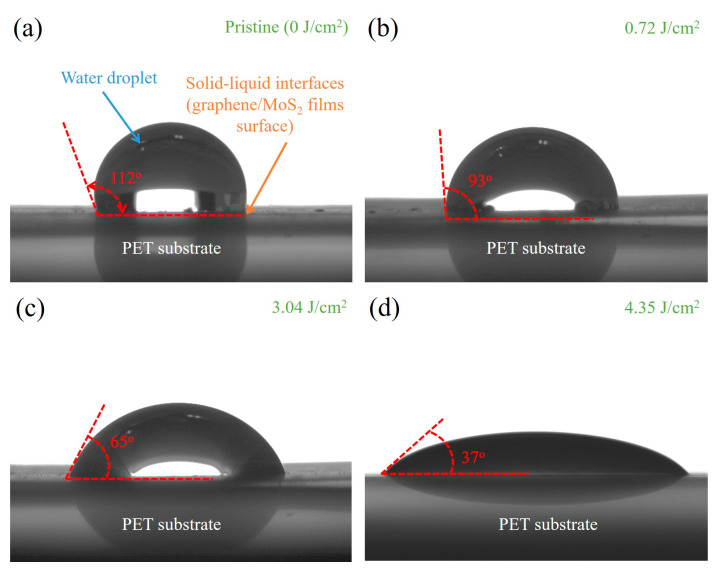
The CCD images of water droplets on graphene/MoS_2_ composite films processed at different laser fluences for contact angle analysis: (**a**) pristine (0 J/cm^2^); (**b**) 0.72 J/cm^2^; (**c**) 3.04 J/cm^2^; (**d**) 4.35 J/cm^2^.

Figure 8 shows the static contact angle CCD images of water droplets on graphene/MoS_2_ composite films processed at different laser fluences. The contact angles progressively decrease from 112° to 37° with increasing laser fluence, indicating a significant enhancement in surface wettability following laser-induced modifications. In the pristine sample (a), the high contact angle of 112° reflects the intrinsic hydrophobic nature of the as-deposited graphene/MoS_2_ composites. Upon a low fluence of 0.72 J/cm^2^, partial removal and surface roughening begin to modify the surface chemistry, reducing the contact angle to 93°. At an optimized fluence of 3.04 J/cm^2^, the contact angle further drops to 65°, suggesting an increase in polar functional groups and structural porosity, both of which promote water adsorption. Under the highest applied fluence of 4.35 J/cm^2^, the surface exhibits nearly complete wetting of 37°, attributed to full ablation of hydrophobic carbonaceous components and formation of a micro-nanostructured oxide-enriched surface. This enhancement in hydrophilicity is consistent with the blue-shifting behavior observed in Raman spectra (see Figure 7), particularly in the *A_1g_* mode of the MoS_2_ and the *G* band of graphene. These shifts are indicative of increased surface strain, interlayer compaction, and possibly hydration-induced lattice distortion, all of which contribute to greater solid–liquid interaction at the sensor interface. The laser-induced transition from hydrophobic to hydrophilic behavior is thus critical for improving electrolyte wetting and ion accessibility, which directly impacts the electrochemical sensitivity of pH sensors based on graphene/MoS_2_ composite electrodes. Similarly, the structural integrity and thermal stability of graphene/MoS_2_ composites have been extensively validated in previous studies. Transmission electron microscopy (TEM) analyses have revealed the formation of layered heterostructures [28], while thermogravimetric (TG) analyses confirmed thermal stability up to approximately 500 °C [38], and X-ray photoelectron spectroscopy (XPS) provides detailed insights into elemental composition and chemical states [50]. These findings align with the focus of the present study on the functional performance of the device, where Raman spectroscopy (see Figure 7 and Figure 8) further substantiates the retention of key structural features in the composite films after laser ablation.

### 3.3. Electrical Characteristics Analysis and pH Detection Sensitivity

**Figure 9:**
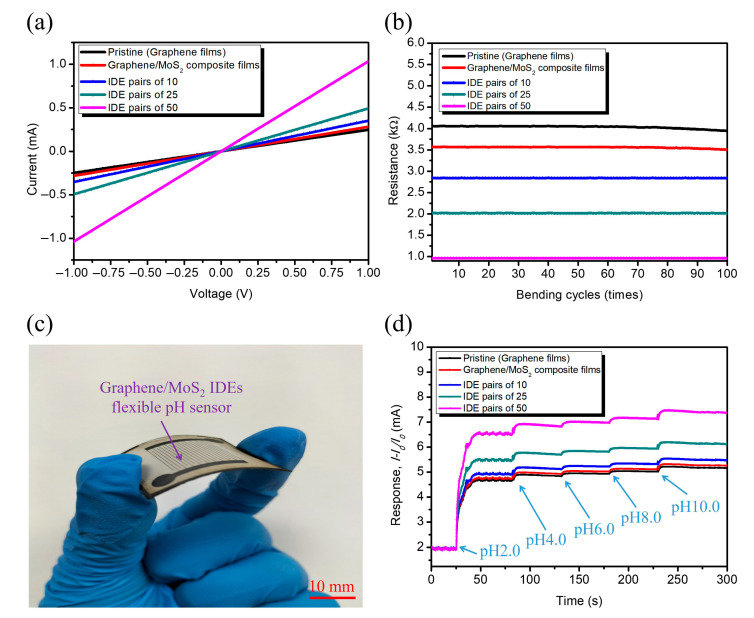
Comparative electrical characterization of pristine graphene films, and graphene/MoS_2_ composite IDE configurations: (**a**) *I–V* curves; (**b**) mechanical durability; (**c**) photograph of structural integrity and mechanical compliance; (**d**) the real-time response to stepwise additions of pH solutions ranging from pH 2.0 to pH 10.0.

To evaluate the electrical and sensing performance of the fabricated graphene/MoS_2_ IDEs flexible pH sensors, a systematic characterization was conducted using pristine graphene films, graphene/MoS_2_ composite films, and graphene/MoS_2_ composites with varying IDE pairs configurations (10, 25, and 50 pairs), as shown in Figure 9a–d. Figure 9a shows the current–voltage (*I–V*) characteristics of each electrode configuration under a voltage sweep from −1.0 V to +1.0 V. The pristine graphene films exhibited a relatively low current response due to limited intrinsic conductivity. In contrast, the incorporation of MoS_2_ particles into the graphene matrix significantly enhanced electrical conductivity, which is attributed to the formation of heterointerfaces that promote charge carrier mobility. Notably, as the graphene/MoS_2_ IDE pairs increased from 10 to 50, the slope of the *I–V* curve became steeper, reflecting reduced resistance and more efficient charge collection. This trend confirms that increasing IDEs density provides a greater interfacial area and stronger local electric fields, which facilitate electron transport across the sensing surface. Figure 9b illustrates the mechanical durability of the different electrode configurations assessed through 100 cycles of repeated bending. The resistance remained stable across all samples, with the 50-pair IDEs demonstrating the lowest and most consistent resistance throughout. The sensor maintained a stable response to pH changes, with less than 5% variation in current sensitivity, demonstrating its resilience against mechanical strain. This result validates the mechanical flexibility and electrical robustness of the laser-formed graphene/MoS_2_ composite electrodes, essential for wearability. Figure 9c presents a photograph of the graphene/MoS_2_ IDEs flexible pH sensor under bending conditions, highlighting its structural integrity and mechanical compliance. The successful implementation of laser ablation patterning on flexible PET substrates enables conformal integration with curved surfaces, an important requirement for epidermal or implantable sensors. Finally, Figure 9d shows the real-time response (defined as *I* − *I*_0_*/I*_0_) of the sensors in response to stepwise additions of pH solutions ranging from pH 2.0 to pH 10.0. A clear increase in current sensitivity was observed with rising pH levels, particularly in the IDE pairs of 50, which exhibited the highest sensitivity and fastest stabilization across the tested pH range. This trend can be explained by the deprotonation of surface functional groups (e.g., *–OH*, *–COOH*) at higher pH levels, resulting in an increased density of negative charges on the graphene/MoS_2_ surface and enhanced charge transfer kinetics [49,51].

Remarkably, the response exhibited near-linear behavior (*R*^2^ = 0.982) with a sensitivity of ~4.7% per pH unit for the 50-pair IDE configuration, demonstrating effective detection of acidic and basic environments. The denser IDE geometry further amplifies this effect by increasing the number of active sensing sites and reducing the diffusion path lengths for ionic species.

**Figure 10:**
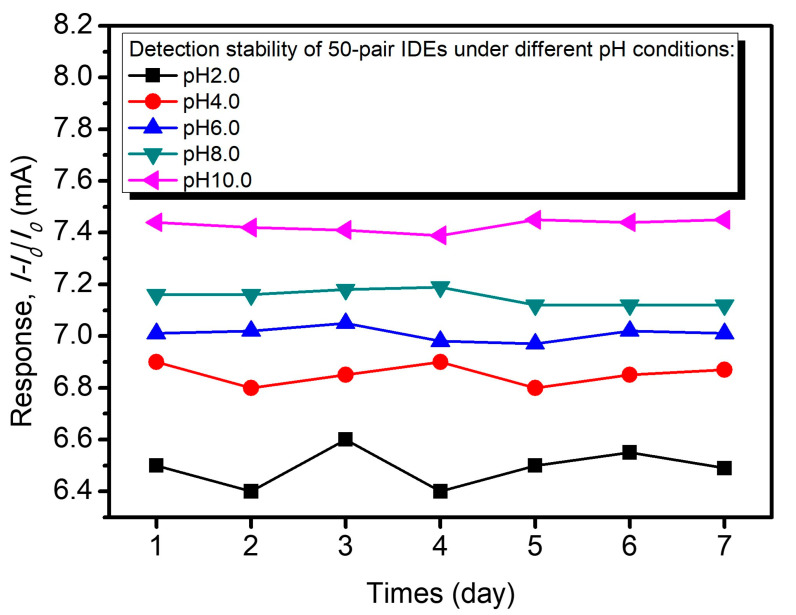
Detection stability of 50-pair IDEs under varying pH conditions over a 7-day period.

To assess the long-term operational stability of the fabricated graphene/MoS_2_ IDE-based pH sensors, the response of the 50-pair IDEs configuration was monitored under five different pH conditions within pH 2.0, pH 4.0, pH 6.0, pH 8.0, and pH 10.0 over a 7-day period, as shown in Figure 10. The results show minimal variation in the response for all pH levels tested, indicating excellent stability (>95%) of the sensor’s electrochemical performance. Notably, at pH 10.0, the response maintained a nearly constant value of ~7.4 mA. These findings demonstrate the robust performance of the laser-ablated graphene/MoS_2_ composite IDEs under repeated exposure to acidic and basic environments. In comparison to previously reported laser-induced graphene pH sensors (sensitivity ~3.2% per pH unit; response time ~10 s) [37], and MoS_2_-based field-effect transistor (FET) sensors (response time ~12 s) [52], the proposed laser-ablated graphene/MoS_2_ IDE-based sensor exhibits a substantial enhancement in both sensitivity (~4.7% per pH unit) and response time (~5.2 s). More importantly, unlike these prior designs that often rely on additional metal passivation layers or complex surface modifications to improve pH responsiveness and wettability [53], this sensor leverages the inherent properties of the graphene/MoS_2_ composite and the precise control of laser ablation to achieve superior performance. This study not only simplifies the fabrication process but also ensures mechanical flexibility and long-term stability, which are critical for wearable and point-of-care diagnostic applications. Collectively, these advantages establish the sensors as a highly promising platform for rapid, reliable, and scalable pH monitoring in dynamic biological environments.

## 4. Conclusions

This work establishes a flexible, laser-ablated graphene/MoS_2_ composite IDE platform that achieves high-performance pH sensing across a wide range from pH 2 to pH 10. Through precise optimization of pulsed laser ablation parameters, particularly at a fluence of 4.35 J/cm^2^, defect-free interdigitated structures were fabricated without compromising PET substrate integrity. The resulting device demonstrated a sensitivity of approximately 4.7% per pH unit and a rapid response time of ~5.2 s, outperforming comparable graphene- and MoS_2_-based sensors reported previously. Notably, the 50-pairs IDE configuration provided the highest current response and exhibited excellent long-term stability, maintaining > 95% of its sensitivity over 7 days of ambient storage and negligible performance degradation after 100 bending cycles. These achievements underscore the synergy between laser micromachining and graphene/MoS_2_ heterostructures in enabling scalable, non-invasive pH sensors for wearable applications. The proposed sensor architecture holds strong potential for integration into point-of-care diagnostic systems and continuous wound monitoring platforms, paving the way for real-time, label-free monitoring in dynamic biological environments.

## Figures and Tables

**Table 1:** Geometric design parameters of three IDE pairs configurations used for pH sensing.

Type	Active Sensing Area (mm^2^)	IDE Width (μm)	IDE Pairs	IDE Pitch (mm)
I	30 × 30	80	10	0.62
II			25	0.18
III			50	0.04

## Data Availability

All the data generated or analyzed during this investigation are included in this article.
